# Review: Proposed Methods to Improve the Survival of Adipose Tissue in Autologous Fat Grafting

**DOI:** 10.1097/GOX.0000000000001870

**Published:** 2018-08-03

**Authors:** Mark J. Landau, Zoe E. Birnbaum, Lauren G. Kurtz, Joel A. Aronowitz

**Affiliations:** From the * Keck School of Medicine of USC, Los Angeles, Calif., 90033; † University Stem Cell Center, Los Angeles, Calif., 90048; ‡ Cedars-Sinai Medical Center, Los Angeles, Calif, 90048.

## Abstract

In 2009, the American Society of Plastic Surgeons Task Force on Autologous Fat Grafting (AFG) determined that autologous fat grafting was a safe procedure with a relatively low rate of complications. This consensus opinion unleashed a wave of popularity as plastic surgeons discovered the procedures' efficacy in a wide variety of cosmetic and reconstructive indications. Frequently reported cosmetic applications include soft-tissue augmentation of breast, buttocks, hips, face, and hands, whereas reconstructive applications include adjunct for breast reconstruction contour problems, plantar fat pad improvement, and correction of various posttraumatic and surgical contour deformities. Recognition of other regenerative effects of fat grafting expanded the use AFG for improvement of hypertrophic scar tissue, postradiation sequelae, lipodystrophy, hyperpigmentation, senile skin changes, and actinic damage. The popularity of AFG is supported by a remarkably low risk of complications, minimal scars, and readily available donor sites. Despite recognition of the advantages of AFG, there still is no consensus regarding optimal techniques of harvest, graft preparation, and injection. Further, the yield of permanent volume falls within a very wide range. In this article, we review the basic science of fat grafting, proposed methods offered to improve engraftment, and reported outcomes of AFG procedures.

## INTRODUCTION

Autologous fat grafting (AFG) is not a new technique. It was first described by Neubar^1^ in 1893 to fill a depressed facial scar. Used only sparingly for the greater part of the next century, AFG was mostly overlooked in plastic and reconstructive surgery until the early 1980’s. In 1987, as the popularity of AFG surged, the American Society of Plastic and Reconstructive Surgeons issued a position paper on the use of autologous fat grafting in the breast. At the time, they did not condone the use of autologous fat grafting on the grounds that “much of the injected fat will not survive and the known physiological response to necrosis of this tissue is scarring and calcification. As a result, detection of early breast carcinoma through xenography and mammography will become difficult.^2,3^”

Since 1987, the popularity of fat grafting for a variety of indications has only increased, and a significant body of literature demonstrated that artifacts resulting from fat grafting, mainly calcifications and oil cysts, can be readily differentiated from malignancy in early breast cancer screening.^4–9^ This is due to improvements in imaging techniques and an expanded body of data on the subject. In 2009, the American Society of Plastic Surgeons published a new position paper evaluating the safety and efficacy of autologous fat grafting. The American Society of Plastic Surgeons task force determined that autologous fat grafting was a safe procedure with a relatively low rate of complications. In addition, they stated that artifacts could indeed be identified and distinguished from malignancy in early breast cancer screenings.^10^

In recent years, AFG has become a widely accepted and utilized technique in the field of plastic surgery due to its application in soft-tissue augmentation and its regenerative effects on local tissue such as reversal of hyperpigmentation, softening of hypertrophic scars, increased local vascularity, and improvement of radiated tissue.^11–13^ The diverse applications of fat grafting within the field of plastic surgery include facial rejuvenation, hand rejuvenation, breast reconstruction and volume enhancement, treatment of skin photoaging, correction of contour deformities, and improvement of senile and diabetic plantar fat pad atrophy.^14–20^ AFG is useful for both cosmetic and reconstructive indications because there is no scarring at the injection site, no foreign material implanted, a low rate of serious complications, and a typically desirable donor site.

Despite the beneficial effects, there are some drawbacks associated with AFG. Postfat grafting soft-tissue lesions such as calcifications and oil cysts are observed postoperatively. But it is clear that lesions appearing more than 1 year after fat grafting are not directly related to the procedure.^14,21^

The most troublesome and persistent issue in fat grafting, however, is the unpredictable degree of volume retention of fat grafts after transplant. Across the literature, studies have reported a wide range of reabsorption rates after transplantation.^22^ Although some of the variability is no doubt due to inconsistent methods of pre- and postoperative volume assessment used in various clinical studies reported, a lack of predictable volume retention is clearly a characteristic of autologous fat grafting. A better understanding of the fat graft microenvironment in which engraftment occurs is clearly critical to improvement of clinical outcomes. The current state of knowledge of fat graft failure points toward cellular hypoxia as the major factor adversely affecting the engraftment process and thus long-term volume retention. Studies demonstrate that mature adipocytes can tolerate hypoxia for approximately 24 hours at normal core body temperature due to the relatively active metabolic demand of their intracellular cytoplasm. In vivo, fat grafting involves placement of a 1–4 mm diameter adipose tissue fragments, each consisting of thousands of individual cells, into a profoundly ischemic adipose tissue recipient site microenvironment. Ideally, the graft fragment is initially nourished by diffusion of oxygen and glucose from the surrounding tissue and quickly revascularized through microvascular inosculation and neovascularization. The clinical success of fat grafting attests to the efficiency of this revascularization process. A significant proportion of injected fat, however, fails to successfully engraft due to irreversible anoxic injury suffered by adipocytes before revascularization can occur. Passive diffusion of oxygen and glucose is not sufficient to sustain adipocytes located centrally in the individual graft tissue fragment, that is, at a depth of more than about 10 cells from the fragment surface and thus anoxia-induced apoptosis and resorption is the fate of the central core of the graft fragment. Improvement in AFG technique is aimed at reducing the number of mature adipocytes located intermediate between the well-perfused outer cell layers and the anoxic central core of the graft fragment that enter an apoptotic pathway and fail to engraft.^23^

Studies have improved understanding of the cellular processes affecting the success of fat grafting and offer the possibility of improving the reliability and predictability of fat grafting. This work, for example, revealed hypoxia-induced factors expressed by adipocytes, which are exposed to ischemic conditions. But the identification of an adipose-specific apoptosis pathway induced by hypoxic conditions and which might be affected by changes in operative technique or medication remains an unrealized research goal. Still, it is possible to identify several factors that contribute to high rates of adipocyte apoptosis and ultimately fat graft resorption. These factors include the threshold parameters tolerated by grafted cells to specific hyponutritional microenvironments, anoxia, and physical trauma, which occurs before revascularization. The clinical value of this knowledge is supported by studies that show administration of proangiogenic factors such as erythropoietin and vascular endothelial growth factor improve the survival of transplanted fat tissue in mouse models.^24,25^ Another salient factor in successful engraftment is the presence of adipose-derived stem cells (ASCs). These cells are pluripotent mesenchymal stem cells that reside in large numbers in adipose tissue. They are concentrated in the stromal vascular fraction (SVF) of lipoaspirate. These small stellate-shaped cells are identified by surface antigens such as CD134 and their ability to form colonies in vitro. It is estimated that 1–3 million of these small stellate-shaped cells typically reside in proximity to small vessels of adipose tissue. Adipose stem cells are known to tolerate the conditions associated with harvest and graft injection more successfully than mature adipocytes, participate in the tissue response to these stresses, and direct adipose tissue regeneration. Importantly, the liposuction fat graft harvest process depletes the number of ASCs in fat graft material.^26,27^

These observations, and other factors known to affect successful engraftment, suggest several strategies that may be expected to improve volumetric retention by optimizing the processes of fat graft harvest, graft preparation, and the fat graft injection. The most salient factors amenable to adjustment are fat graft particle size, graft physical trauma, graft hypoxic time, number of ASCs in the fat graft material, and the recipient microenvironment. In this article, we review proposed methods to improve the effectiveness of AFG, the relevant basic research basis of the strategy, and the results of the relevant clinical research.

## HARVEST, HANDLING, AND GRAFTING TECHNIQUE

Many groups have attempted to determine the optimal techniques for harvest, processing, and transplantation, but there is still no general consensus on the most effective technique. This lack of consensus regarding technique contributes to the wide range of graft retention rates reported across the literature. Part of the problem stems from the fact that AFG actually consists of a multiplicity of individual steps from fat harvest, to graft preparation, to fat injection. Most of the component steps at each stage are likely to substantially affect survival of each small adipose tissue fragment, which constitutes the injected graft, thereby affecting the permanent graft volume. Compounding the problem of controlling these multiple variables in clinical studies is the difficulty of studying the major end point, that is, volume retention. Volumetric measurements, although simpler today with more advanced photographic methods, are notoriously difficult and results are often difficult to compare due to significant differences in both the harvest and recipient site. Nevertheless, there is a significant body of knowledge concerning the factors known to affect graft viability and the engraftment process. These factors fall under graft harvest methods, fat graft processing methods, and grafting technique. They are reviewed below.

### Fat Harvest Methods

In 2013, Fisher et al.^28^ investigated fat harvest techniques used in AFG. They compared fat harvest using either suction-assisted liposuction or ultrasound-assisted liposuction. In terms of graft retention and SVF content, there was no significant difference between the 2 methods. This was reinforced by Chung et al.^29^ in 2014 who reported no decrease in viability with ultrasound-assisted or suction-assisted liposuction; however, when comparing suction-assisted liposuction with laser-assisted liposuction, they noted that laser-assisted liposuction reduced viability of graft material.

Another factor that was proposed as a point of variance in producing lipoaspirate graft material is the location of harvest. Numerous studies have investigated fat graft characteristics and AFG results based on harvest location. In 2004, Rohrich et al.^30^ examined fat harvested from 4 body parts: the abdomen, the flank, the thigh, and the medial knee. They determined that there was no significant effect on adipocyte viability or survival based on harvest site. In general, harvest location has not been determined to not be a significant factor in the outcomes of AFG procedures. This information is beneficial because it allows the surgeon more versatility to harvest based on tissue availability and patient aesthetic preferences without compromising the outcomes.

### Fat Processing Methods

Fisher et al.^28^ also compared 3 common processing techniques: filtration, cotton gauze rolling, and centrifugation. In the filtration method, lipoaspirate is passed through an 800 µm filter. In the centrifugation method, often referred to as the Coleman technique, lipoaspirate is centrifuged at 3,000 rpm for 3 minutes. The adipose layer is then separated from the aqueous and oil layers. In the cotton gauze rolling method, fat is gently rolled on a nonadherent cotton gauze dressing for 5 minutes using a sterile scalpel to remove the liquid portions. When grafted, the cotton gauze method was reported to have the highest volume retention compared with the other methods, with 70% retention. The filtration method retained 58% and the centrifugation method retained only 47%. Fisher et al.^28^ suggested that cotton-gauze was the preferred method for cosmetically sensitive parts of the body where less fat is required, but filtration and centrifugation were more practical options for large volumes.

### Grafting Technique

No strict recommendation can be made about the best processing technique for AFG based on the literature. Various individual studies have found different methods to be superior depending on the protocols used in each study.^31^ However, there are common trends and observations reported across many studies. One observation is that grafts prepared using simple decantation contained the highest number of viable adipocytes, but also the highest number of contaminants. Additionally, grafts prepared using centrifugation at a speed above 400 g have decreased viability of adipocytes.

## PLATELET RICH PLASMA

A method receiving a significant amount of attention for its potential to improve volumetric retention of fat grafts is the supplementation of graft tissue with autologous platelet-rich plasma (PRP). PRP is a growth factor–rich injectable that can be generated quickly and cost effectively from a patient’s own blood. Over 800 different proteins have been identified to be secreted by platelets into the plasma, which affect a wide range of cells in the body.^32^

Platelets are a vital part of the immune system response to endothelial injury. Platelets, normally inactive, become activated when they come in contact with damaged endothelial tissue and can also be quickly activated in vitro by contact with glass, freezing cycles, or the addition of calcium or thrombin. Once activated, platelets release stores of growth factors that facilitate tissue repair. Growth factor secretion is most intense in the first hour but continues for about 7 days.^33^ The growth factors synthesized by platelets stimulate healing and tissue repair through intercellular mediators and cytokines that stimulate angiogenesis and promote cell proliferation, cell differentiation, and extracellular matrix formation.^34–36^ Although synthetic forms of these growth factors have been previously studied, they are more readily available and more easily acquired for clinical use in the form of autologous PRP. PRP has been used over the last 30 years to promote bone regeneration, wound healing, tendon and cartilage repair, corneal repair, and skin rejuvenation.^37–42^ It should, however, be noted that results across different applications of PRP can sometimes be difficult to compare. This is because since the 1970’s a variety of different methods have been used to prepare PRP, leading to significant variation in the composition and outcomes.^43,44^

In terms of improving fat grafting, PRP has multiple potential beneficial qualities. The growth factor stores in PRP allow cells to resist the hypoxic stress experienced within the first few days after fat transfer and promote proper arrangement of transplanted tissue by facilitating production of the extracellular matrix. The growth factors in PRP also promote angiogenesis, which facilitates recovery from the ischemia associated with fat transfer. PRP has been shown to improve fat survival rate and promote stem cell proliferation and differentiation in vitro.^45,46^

Numerous animal studies have shown a positive relationship between PRP-enhancement and fat graft survival. A study published by Nakamura et al.^47^ in 2010 demonstrated that fat grafts enhanced with PRP retained significantly more volume than nonenhanced fat grafts in rats. A similar result was achieved by Pires Fraga et al.^48^ in 2010 using rabbits, demonstrating greater volume retention and blood vessel formation and less necrosis and fibrosis. A study by Rodríguez-Flores et al.^49^ in 2011 compared the histological characteristics of grafted tissue with and without PRP in rabbits. They noted that when PRP was included, there was less inflammation observed in the recipient site, fewer instances of oil cyst formation, and increased survival of transplanted fat cells.

Recently, additional studies have examined clinical applications of PRP-enhanced fat grafting for wound healing, facial reconstruction, and general aesthetic improvements.^50^ Studies have demonstrated that the application of SVF and PRP have similar effectiveness in the treatment of posttraumatic lower extremity ulcers and facial scars.^51,52^

In 2012, Gentile et al.^53^ published clinical results of a study comparing PRP-enhanced AFG and normal AFG in breast reconstruction. They observed that after 1 year, the PRP-enhanced group (n = 50) retained 69% of the initial 3-dimensional volume, whereas the control group (normal AFG, n = 50) retained only 39%. In 2013, the same group published another article expanding on their results with the procedure they termed Platelet-rich Lipotransfer.^54^ They observed that when using a PRP concentration of 0.5 mL or 0.4 mL of PRP per mL of fat tissue, 70% of the initial volume was retained at 1 year, compared with only 31% in the control group.

## RECIPIENT SITE IMMOBILIZATION WITH NEUROTOXIN

An interesting approach proposed by Baek et al.^55^ in 2012 suggested that enhancement of fat grafts with Botulinum Toxin A (BoNTA) could improve fat graft survival in the facial region, and they tested this theory in a rat model. Fat was excised from the rat retroperitoneal area and then digested with collagenase for 2 hours to create a homogenate. This homogenized fat mixture was then centrifuged to remove the fluid components and used for grafting. In each rat, Baek et al.^55^ grafted 2 separate tissue deposits, 1 on each side of the back. One of these grafts was injected with fat, saline and BoNTA (0.5 IU), whereas the other received only fat and saline. Baek et al.^55^ observed a significant increase in the weight, volume, and cellular integrity of the graft which received BoNTA compared with the control graft. They reported 74% volume retention of the initial 0.5 mL of fat tissue grafted in the BoNTA group and only 44% in the control group.

The authors proposed that the increased retention was due to temporary muscle immobilization provided by the BoNTA, which decreases abnormal muscle contractions in the face and preserves graft viability. For the cosmetic treatment of facial aging, BoNTA injections to the face are commonly performed shortly before or after facial fat grafting procedures are conducted.^56^ This method proposed by Baek et al.^55^ seeks to consolidate these 2 into 1 procedure for patient convenience and potentially improved results. However, more clinical evidence is required to validate this technique in humans.

## ADIPOSE-DERIVED STEM CELLS AND CELL-ASSISTED LIPOTRANSFER

In 2006, the research team led by Yoshimura et al. published an article in which they described a method of supplementing the lipoaspirate used for fat grafting with progenitor cells found in adipose tissue, adipose-derived stem cells (ASCs).^58^ They termed this process cell-assisted lipotransfer (CAL).^57^ The rationale behind this technique is that aspirated adipose tissue (lipoaspirate) is generally poor in progenitor cells, which is a contributing factor to poor survival in vivo. Lipoaspirate is poor in progenitor cells for 2 main reasons. The first is that ASCs tend to be located closer to major blood vessels in adipose tissue which are avoided during liposuction and other harvest techniques.^26,27^ The second reason is that a portion of the progenitor cells are contained in the fluid portion of the lipoaspirate, which is discarded before grafting.^58^ To combat this problem, Yoshimura et al. suggested harvesting excess lipoaspirate and isolating the progenitor cells contained within, which then can be used to supplement the lipoaspirate to create a progenitor-rich graft.^26,27,58^

Adipose-derived stem cells have many characteristics which aid in the retention of fat grafts. First, ASCs are able to differentiate into new adipocytes, replacing a portion of the adipocytes, which succumb to apoptosis due to hypoxic or physical stress.^59–61^ Second, ASCs have been shown to actively promote angiogenesis via growth factor secretion and through neovascular differentiation.^62,63^ ASCs have been shown to not only survive in hypoxic conditions, but actually significantly increase their production of soluble angiogenic growth factors, including vascular endothelial growth factor and hepatocyte growth factor.^64,65^ By promoting the development of new vasculature in the grafted tissue, ASCs are able to speed the recovery from ischemia after transplantation and reduce the number of cells succumbing to hypoxic stress, thereby improving graft volume retention.

Studies have been conducted using both the SVF and pure, cultured populations of ASCs. SVF is a heterogeneous population of cells that results from the processing of adipose tissue and is composed mainly of various blood cells, pericytes, macrophages, smooth muscle cells, and both adipose-derived and vascular endothelial progenitor cells.^66^ The use of a pure population of ASCs was not shown to be superior to using SVF cells. Using SVF cells is advantageous because they do not require culturing, which can take weeks. Instead, these cells can be isolated and injected in the same surgical procedure. Using cultured cells would require 2 procedures: 1 to harvest cells and 1 for the actual grafting. In the initial article describing CAL, they reported a 35% increase in retention of grafted tissue volume compared with normal fat grafting in a mouse model. This has led to many projects investigating the therapeutic potential of CAL. In 2013, Kølle et al.^67^ conducted a randomized placebo-controlled trial to investigate the effects of ASC enhancement on graft survival in humans. Using cultured ASCs, Kølle et al.^67^ reported significantly higher levels of volume retention compared with controls. The ASC-enhanced group retained 80.9% of the initial volume on average, compared with the control group which only retained 16.3% on average. Another study by Wang et al.^68^ published in 2012 reported CAL results from 18 patients. They reported retention of only about 50% of the grafted tissue at 6 months. Although there is still absorption of a significant portion of fat after grafting, studies on CAL have reported a positive correlation with ASC enhancement and volume retention compared with normal AFG, but how much the retention is improved is still debated, and no conclusive dose versus effect relationship has been established.^69^

The main application of CAL in the clinical setting has been for cosmetic breast augmentation and reconstruction, but with these recent advances in the field of AFG, essentially all previous applications of fat grafting are now being reinvestigated using CAL, including the treatment of lipodystrophy and contour defects, reduction of facial aging, and accelerated healing of chronic wounds, among others.

## CAL AND PRP IN COMBINATION

The independent advances observed with both PRP and ASC enrichment of fat grafts naturally led to the attempt to combine the 2. PRP has been shown to increase the proliferation and differentiation of ASC in vitro.^70,71^ By supplementing ASC enhanced fat grafting (CAL) with PRP, researchers and clinicians hope to boost the regenerative effects of the stem cells, while still getting the benefit afforded by PRP enhancement, to achieve a method superior to supplementation with either alone.

In a recent study by Seyhan et al.,^72^ each of these 3 methods (ASC only, PRP only, and ASC + PRP) were compared in rats. They reported that after 12 weeks the PRP + ASC group had the highest weight and volume of fat grafts while also having the highest number of viable adipocytes and blood vessels. Growth factor levels were also the highest in the PRP + ASC group. While in vitro and animal studies are promising, there is relatively no clinical data available on the combination of the 2, most likely because of the lack of adequate clinical data on the use of ASCs alone. Studies have shown that the injection of ASC combined with PRP accelerated wound closure rates in patients with chronic skin ulcers, but the individual contributions of ASC and PRP to wound closure and their possible synergism has not yet been elucidated.^73^ There are also studies which examine the combination of PRP and ASCs in areas outside of fat grafting, such as improvement of knee joint function.^74^

## CONCLUSIONS

Fat grafting continues to increase in popularity with new indications and novel technical modifications reported frequently. AFG helps augment and regenerate deficient, scarred, irradiated and aged subcutaneous soft tissue, and skin in a wide variety of clinical situations with a low complication rate and low donor-site morbidity. However, there is still no clinically vetted ideal technique that ensures maximum graft survival and predictability of lasting graft volume. There is currently no consensus on the optimal autologous fat grafting technique that produces the most predictable outcome for a given clinical situation. Unlike most operations, successful fat engraftment does not occur at the macroscopic tissue level, and tissue viability can not be assessed by more familiar parameters such as color, warmth, tension, and bleeding. Now plastic surgeons are compelled to delve to the level of very small tissue fragments and even individual cells which are affected by such novel factors as local oxygen tension, glucose concentration, micro physical stress, ambient temperature effects on cellular metabolism, and the like. But just as improved tissue rearrangement procedures using muscle flaps emerged from an improved understanding of the axial blood supply, it is expected that improved AFG outcomes will result from meticulous attention to the factors which affect viability of small fat tissue fragments and cells.

Studies have been conducted to examine if using PRP, ASC, or a combination of both will help combat the low fat graft retention rates, but no ideal method has been determined. Although there is some anecdotal evidence, more clinical evidence is required to validate the best technique to ensure the volumetric retention of adipose tissue after autologous fat grafting. Future research will hopefully refine our understanding of the effect of fat grafting on the local tissue micro-environment and provide clues toward its optimization. Correlating laboratory results with large-scale, controlled studies using human patients is needed to advance our ability to tailor fat grafting techniques to specific medical and cosmetic applications. Based on the results of cell biology research and the long-term accumulation of objective patient data, we believe that a standard technique of fat grafting for a given clinical scenario will emerge.
